# Brain Citrullination Patterns and T Cell Reactivity of Cerebrospinal Fluid-Derived CD4^+^ T Cells in Multiple Sclerosis

**DOI:** 10.3389/fimmu.2019.00540

**Published:** 2019-04-10

**Authors:** Wolfgang Faigle, Carolina Cruciani, Witold Wolski, Bernd Roschitzki, Marco Puthenparampil, Paula Tomas-Ojer, Carla Sellés-Moreno, Thomas Zeis, Ivan Jelcic, Nicole Schaeren-Wiemers, Mireia Sospedra, Roland Martin

**Affiliations:** ^1^Neuroimmunology and MS Research Section, Neurology Clinic, University Zurich, University Hospital Zurich, Zurich, Switzerland; ^2^Functional Genomics Center Zurich, ETH Zurich & University of Zurich, Zurich, Switzerland; ^3^Neurobiology, Department of Biomedicine, University Hospital Basel, University of Basel, Basel, Switzerland

**Keywords:** human brain, multiple sclerosis, citrullination, T cell reactivity, autoimmune, proteomics

## Abstract

Immune responses to citrullinated peptides have been described in autoimmune diseases like rheumatoid arthritis (RA) and multiple sclerosis (MS). We investigated the post-translational modification (PTM), arginine to citrulline, in brain tissue of MS patients and controls (C) by proteomics and subsequently the cellular immune response of cerebrospinal fluid (CSF)-infiltrating T cells to citrullinated and unmodified peptides of myelin basic protein (MBP). Using specifically adapted tissue extraction- and combined data interpretation protocols we could establish a map of citrullinated proteins by identifying more than 80 proteins with two or more citrullinated peptides in human brain tissue. We report many of them for the first time. For the already described citrullinated proteins MBP, GFAP, and vimentin, we could identify additional citrullinated sites. The number of modified proteins in MS white matter was higher than control tissue. Citrullinated peptides are considered neoepitopes that may trigger autoreactivity. We used newly identified epitopes and previously reported immunodominant myelin peptides in their citrullinated and non-citrullinated form to address the recognition of CSF-infiltrating CD4^+^ T cells from 22 MS patients by measuring proliferation and IFN-γ secretion. We did not detect marked responses to citrullinated peptides, but slightly more strongly to the non-modified version. Based on these data, we conclude that citrullination does not appear to be an important activating factor of a T cell response, but could be the consequence of an immune- or inflammatory response. Our approach allowed us to perform a deep proteome analysis and opens new technical possibilities to analyze complex PTM patterns on minute quantities of rare tissue samples.

## Introduction

Citrullination is one of more than 400 known PTMs in human proteins. The enzyme, peptidylarginine deiminase (PADI), is responsible for modifying the amino acid arginine to the amino acid citrulline. PADI is part of an enzyme family with five known members (1), and each of these shows a distinct tissue and substrate specificity. The enzymatic reaction results in the loss of a positive charge of the peptide fragment and a mass increase by 1 Da. The process of deimination is considered irreversible since no citrulline-iminase is known so far (2)_._ Gudman and colleagues described citrullination in the context of diseases and reported and postulated an increase of citrullinated proteins in all inflammatory diseases (3). The effects of citrullination on protein function depend on the location of the protein and the position of the amino acid arginine. Since it removes a positive charge from arginine, it may loosen protein interactions and render them more prone to denaturation and degradation (4, 5). Citrullination plays a role in several physiological mechanisms like skin keratinization, myelin formation/remyelination, gene regulation and immune functions. In specialized cells like neutrophils, histone hypercitrullination is an essential process in the formation of highly decondensed chromatin structures termed neutrophil extracellular traps (NETs), which enable these cells to trap and kill bacteria. During the last 10 years, great attention has been paid to citrullination because of its role in inducing anti-citrullinated proteins/peptide antibodies (ACPA) (6). Involvement of citrullination in various diseases like rheumatoid arthritis (RA), multiple sclerosis (MS), psoriasis, chronic obstructive pulmonary disease (COPD), and Alzheimer has been reported (7). In RA, immune reactivity toward various citrullinated self-proteins and self-peptides like fibrinogen, vimentin, fibrin, collagen type II, α-enolase and its involvement in the disease pathogenesis have been well-established (8).

In MS, a chronic inflammatory demyelinating autoimmune disease of the central nervous system (CNS), the amount of the citrullinated myelin sheath protein myelin basic protein (MBP) is increased in white matter as compared to control brains (9), although these findings remain controversial (10, 11). In MBP that has been purified from MS brain tissue, citrullination of six of the nineteen arginines has been found. Among the myelin components, MBP has been studied in greatest detail due to its importance for inducing experimental autoimmune encephalomyelitis (EAE), a rodent model for MS (12). The identification of CD4^+^ T cells reactive against epitopes of several myelin proteins has been a consistent finding (12). We had previously described reactivity of peripheral blood T cells against post-translational modifications of autoantigens, specifically against citrullinated peptides, in MS patients (13). These preliminary studies hinted at elevated T cell reactivity against citrullinated MBP and indicated that T cells specific for citrullinated epitopes could escape central immune tolerance (13, 14).

In the last years the focus has shifted from peripheral blood-derived T cells to those that are found within the CNS compartment, i.e. in the brain and cerebrospinal fluid (CSF) (15, 16), since CNS-infiltrating T cells are considered more likely to be relevant than those from the peripheral blood due to their infiltration of the target tissue. Besides MBP, few other proteins including glial fibrillary acidic protein (GFAP), neurogranin, and histone H3 have been described to be citrullinated in MS brain (10, 17).

## Material and methods

### Human Brain Tissue Preparation

#### Tissue Collection

The UK Multiple Sclerosis Tissue Bank (UK Multicenter Research Ethics Committee, MREC/02/2/39 and KEK-ZH-Nr. 2014-0243), funded by the Multiple Sclerosis Society of Great Britain and Northern Ireland (registered charity 207495) supplied all the tissue samples. Tissue samples from white- and gray matter were isolated from 9 control and 15 MS cases. Gray matter samples were from 6 controls and 6 MS cases, white matter samples from 3 controls and 9 MS cases. All brains have been screened by a neuropathologist to confirm the diagnosis of MS and to exclude other confounding pathologies (UK MS Tissue Bank).

#### Immunohistochemistry

All tissues were analyzed by immunohistochemistry. In order to differentiate between white and gray matter, we stained the tissue with anti-myelin oligodendrocyte glycoprotein antibodies (MOG) and Luxol fast blue (LFB) for myelin as well as anti-HLA-DR for macrophages/microglia. Regions of gray matter, white matter, as well as lesions with active inflammation, areas of remyelination, and demyelinated lesions without active inflammation, could be identified. For LFB staining, LN3 (anti-HLA-DR), and MOG cryostat sections (12 μm) were fixed for 10 min in 4% para-formaldehyde (PFA). Endogenous peroxidase was blocked with 0.6% hydrogen peroxide in PBS or 80% methanol for MOG staining. MOG staining was further delipidated in 100% methanol at −20°C for 8 min. Tissues were blocked with blocking buffer 1% normal donkey serum (NDS), 0.1% Triton, 0.05% Tween in PBS, LN3: 5% NDS, 1% fish skin gelatin 0.3 M glycine in PBS) and incubated with the primary antibodies at 4°C overnight. Secondary biotinylated antibodies were applied for 2 h at room temperature followed by the ABC complex reagent (Vector Labs, Burlingame, California, USA) for 1 h. The color reaction was performed with 3-Amino-9-ethylcarbazole (18). For some sections, counterstaining in hematoxylin was applied for 1 min followed by rinsing the slide in running tap water. For citrulline staining fresh frozen tissue sections were first air-dried for 20 min. before fixing them in methanol at −20°C. Incubation of sections with PBS before and blocking in PBS/10%BSA for 1 h at RT. Incubation with antibody F95 was performed overnight at 4°C. Secondary biotinylated antibody was applied for 1 h at RT followed by the ABC complex reagent (VectorLabs) for 1 h. The color reaction was performed with “ImpactDAB” (VectorLabs) and counterstaining with hematoxylin as described above. Antibodies: LN3 (Abcam, ab190298, 1:250), anti-MOG (Clone Z12, 1:100), anti-citrulline F95 (Millipore, Burlington, Massachusetts, USA). LFB staining was done with cryostat sections (12 μm), fixed for 10 min in 4% PFA. Sections were washed in PBS (3 × 5 min) and ddH_2_O (2 × 1 min), then incubated in 50% ethanol (5 min), 70% ethanol (5 min), 80% ethanol (5 min), and 96% ethanol (5 min). Sections were incubated in 0.1% LFB solution for 20 h at 56°C. Sections were washed in ethanol and ddH_2_O and developed in lithium carbonate (0.05% in ddH_2_O) for 15 s and 70% ethanol for 1 min at RT. After cresyl violet staining (4 min, RT) sections were differentiated in 96% ethanol (30 s), dehydrated in ethanol and xylol and finally mounted with “Entellan” (Merck Millipore).

#### Protein Extraction Protocol

**Figure 1:**
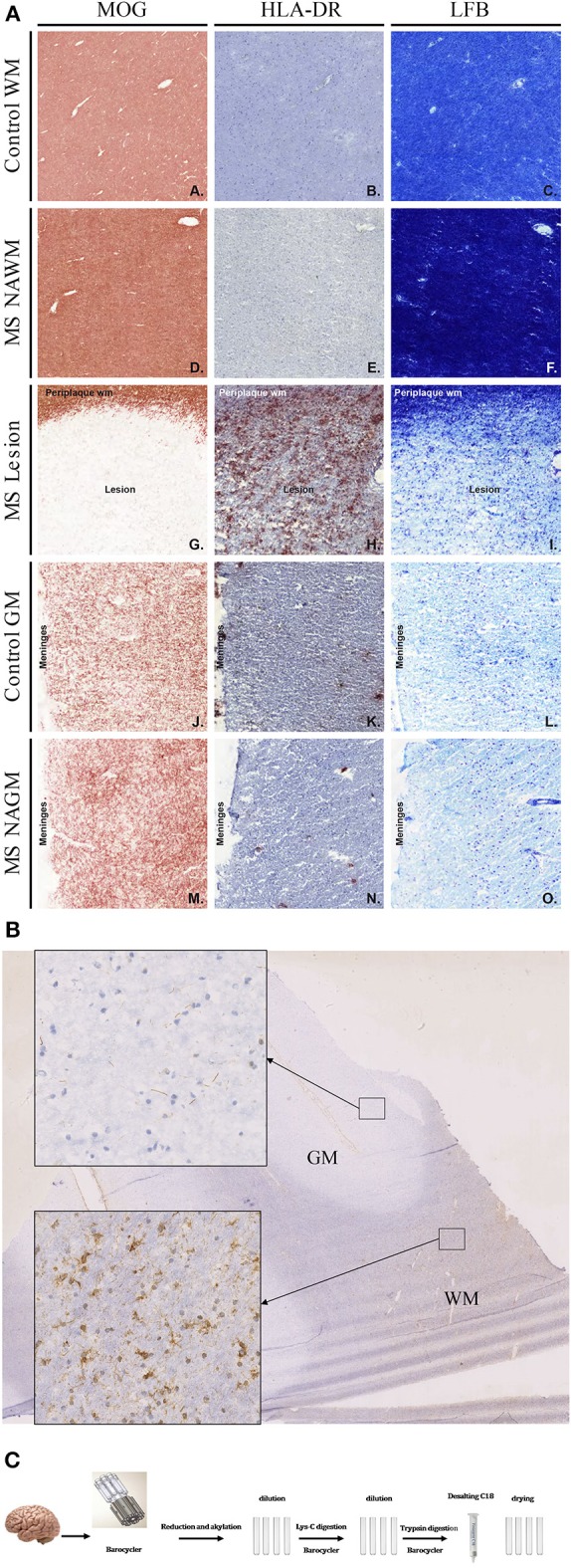
**(A)** Histopathological examination of control and MS tissue. To identify normal as well as lesioned tissue, tissue blocks containing white as well as gray matter tissue were stained for MOG, HLA-DR, and Luxol Fast-Blue. White and gray Matter control tissue showed no sign of demyelination and inflammation (A–C and J–L). Also, NAWM and NAGM did not show any signs of demyelination or inflammation (D–F and M–O). However, MOG as well as LFB staining clearly show demyelination in lesional areas (G and I). Further, a strong HLA-DR staining was visible in lesion tissue (H). **(B)** Citrullination pattern in WM and GM of MS brain (staining with anti-citrulline antibody F95). Inserts showing detail view of corresponding tissue. **(C)** 2–3 mg of brain tissue was disrupted in barocycler and digested by Lys-C and trypsin. All treatments were performed in barocycler instrument. Finally, peptides were desalted and dried.

We analyzed gray and white matter tissue from post-mortem human brains (control and MS patients) in order to establish a spectral library and protein database of the main protein constituents of the CNS. The characterization of the tissue used for the analysis is illustrated in Figure 1A. An overview of samples that were used in the analysis together with medical records is listed in Supplementary Table 1. For protein extraction, a barocycler (Barocycler 2320EXT, Pressure BioSciences, Inc, South Easton, MA, USA) was used. Tissue samples of the size of a needle-head (2–3 mg) were put into barocycler 150 μl micro tubes. The tubes were filled with 30 μl of lysis buffer (8 M urea and 0.1 M ammonium-bicarbonate) and complete protease inhibitor cocktail (Roche, Basel, Switzerland) and closed with 150 μl microcaps. After pre-heating the barocycler for 30 min at 33°C, run cycles were performed at 45 kpsi for 60 cycles and 1 min each. Each cycle lasted 50 s at high pressure and 10 s at ambient pressure.

Reduction and alkylation were carried out as follows: Mixing of TCEP [tris (2-carboxyethyl) phosphine] 2.5 mg and IAA (iodoacetamide) 3.7 mg in 50 μl of lysis buffer. A volume of 4.9 μl of this buffer was added to each tube, incubated at 25°C in a thermomixer while shaking at 1,000 rpm and protected from light. By adding lysis buffer, the urea concentration was diluted from 8 to 6 M. Lys-C enzyme digestion was applied to an enzyme to substrate ratio of 1:40. Lys-C (mass spectrometry grade, Wako, Richmond, VA, USA 20 μg/μl) was dissolved in “Milli-Q-water” to a final concentration 4 μg/μl. Digestion was performed in barocycler with 20 kpsi for 45 min. and cycles of 50 s duration at high pressure and 10 s ambient pressure.

A further dilution of urea from 6 to 1.6 M with 0.1 M ammonium-bicarbonate buffer was necessary to achieve trypsin digestion conditions. Trypsin (sequencing grade modified, Promega, Madison, WI, USA) was added to an enzyme to substrate ratio 1:20. The digestion took place for 90 min at 37°C in a barocycler at 20 kpsi and cycle periods of 50 s at high pressure and 10 s at ambient pressure (19). Finally, the solution was transferred to a 1.5 ml Eppendorf tube. The volume was adjusted to 1 ml by adding a 0.1%TFA/3%ACN solution and the reaction was stopped by adding 10% TFA and pH adjusted to a value between pH 2 and 3. The peptides were desalted on solid phase extraction columns (C18/Finisterre, Wicom, Heppenheim, Germany) according to manufacturer protocol. The samples were vacuum concentrated in a “SpeedVac” and the peptides re-dissolved in 3% ACN/0.1% formic acid in a volume of 20–50 μl to a final concentration of 1 μg/μl. Peptide concentration was measured with Nanodrop instrument (Nanodrop 1000, spectrophotometer (Thermo Scientific, Wilmington, DE, USA) and a solution of a concentration of 0.5 μg/μl prepared.

#### Hydrophilic Interaction Liquid Chromatography (HILIC)

Pools of peptide samples of control tissue (C), as well as the MS tissues from gray and white matter were prepared (300 μg each) and separated using Hydrophilic Interaction Liquid Chromatography (HILIC) (Agilent LC1200 equipped with a column Polyamin II 250 × 3.0 mm 120 Å, 5 μm). The applied gradient was formed of the two solvents: **A**: 75% ACN, 8 mM KH_2_PO_4_ and **B**: 5% ACN, 100 mM KH_2_PO_4_ (pH4.0) for 60 min. Fractions of 1 ml were collected in 27 tubes (detailed protocol as Figure S2). To reduce the number of the samples to be analyzed on the mass spectrometer the fractions were pooled from two tubes. Before injection, samples were purified on “Finisterre SPE” columns (Wicom International, Heppenheim, Germany). Samples of 1 ml were vacuum dried and dissolved in an appropriate buffer, 3%ACN/0.1%TFA. In total 11 fractions for each tissue sample (control and MS of gray and white matter) were prepared. After another vacuum drying, peptides were dissolved in 3%ACN/0.1%FA buffer. Concentrations were measured with Nanodrop instrument and adjusted to 0.25 μg/μl. Reference peptides (iRT) were added (iRT, Biognosys, Schlieren, Switzerland) to each sample.

#### Data-Dependent Acquisition (DDA) of the HILIC Fractionated Samples

The HILIC fractionated samples, 44 in total, were run on Easy-nLC 1000 linked to an Orbitrap Fusion instrument (Thermo Fisher, Waltham, Massachusetts, USA) on a gradient of 80 min. Column material was ReproSil-Pur, C18, 120 Å, AQ, 1.9 μm (Dr. Maisch GmbH, Ammerbuch Germany) and column dimensions ID 0.075 mm/length 150 mm. Solvent A 0.1% formic acid in water and Solvent B 0.15 formic acid in acetonitrile. 4 μl of the sample at a concentration of 0.25 μg/μl was injected.

### Peptide and Protein Identification of HILIC Samples

#### Identification by “Mascot”

We converted “Raw” files converted into “mgf” (20) files and analyzed them on MASCOT software with a human UniProtKB/Swiss-Prot protein database (date: March 22, 2016 with 40,912 entries): Search parameters were 0.05 Da fragment mass tolerance and 10 ppm precursor mass, minimal number of peptides 2, and FDR (false discovery rate) of 0.1%, allowing 2 mis-cleavages on trypsin fragments. We set carbamidomethyl at cysteine as a static modification and oxidation of methionine, deamidation on arginine (R) (with or without neutral loss), glutamine (Q) and asparagine (N) as variable modification. To estimate FDRs separately for deamidated and all the other proteins the mascot.dat files were converted to the bibliospec file format (Skyline).

#### Peptide Identification With “Ursgal”

The universal python module combining bottom-up proteomics tools for large-scale analysis (Ursgal) was used to perform a search with multiple search engines (xtandem vengeance, msgfplus_v9979 and MyriMatch 2 2 140) (21). Evaluation and post-processing of the search results were performed using percolator_2_08. We adjusted the do_it_all_folder_wide.py “Ursgal” example script (https://ursgal.readthedocs.io/en/latest/example_scripts.html#do-it-all-folder-wide) to our input data. We set the variable and fixed modifications in the same as for “Mascot.” For instrument settings, we used the Q-Exactive+ Ursgal profile. We used the “unified_percolator_validated.u_merged_accepted.u_merged.csv” tables (PRIDE), generated by “Ursgal” for further analysis.

#### Post-translational Modifications, Citrullination and Local False Discovery Rate (FDR) Determination

For each peptide, we selected the best peptide-spectrum match (PSM) according to the lowest PEP- (percolator) or Mascot Ion score. We computed the peptide FDR using the target-decoy approach (22) implemented in the R-package TargetDecoyFDR prozor (https://github.com/protViz/TargetDecoyFDR; https://github.com/protViz/prozor). The FDR was estimated separately for the deamidated and citrullinated peptide sequences and all other sequences. The FDR for deamidated and citrullinated peptide sequences increased much faster than for all other sequences (see Figure S1) resulting in a more demanding cutoff score for those sequences. We deposited the mass spectrometry proteomics data in the ProteomeXchange Consortium via the PRIDE (23) partner repository with the dataset identifier PXD008344.

### Isolation, Expansion, and Proliferative Testing of T Cells

#### Peripheral Blood Mononuclear Cell Isolation

Allogeneic peripheral blood mononuclear cells (PBMCs) where isolated freshly from anonymized buffy coats obtained from the Blood Bank of the University Hospital in Zurich. Buffy coats were first diluted 1:1 in PBS, and later PBMCs extracted using a Ficoll gradient. Irradiated (45 Gray) allogeneic PBMCs were used during the freezing procedure of the CSF cells and again during the expansion of CSF-infiltrating CD4^+^ T cells where they functioned as feeder cells. Fresh blood was obtained in EDTA-containing tubes from all patients, from whom CSF samples were available. PBMCs were isolated from fresh blood using Ficoll density gradient centrifugation (PAA, Pasching, Austria) and cryopreserved in 90% FCS (Eurobio) and 10% DMSO (Applichem, Darmstadt, Germany).

#### Isolation and Expansion of CSF-Infiltrating CD4^+^ T Cells

Fresh bulk CSF-derived mononuclear cells from the 22 CIS/RRMS patients were mixed with 5 × 10^6^ allogeneic irradiated PBMCs, and CD4^+^ T cells were subsequently positively selected with anti-CD4 magnetic beads according to the manufacturer's instructions (Miltenyi, Bergisch-Gladbach, Germany). CD4^+^ cell fractions were seeded at 1,500 cells per well in 96-well U-bottom microtiter plates together with 1.5 × 10^5^ allogeneic irradiated PBMCs, 1 μg/ml of PHA-L (Remel, Thermo Fisher, USA) and IL-2 supernatant, derived from the IL-2t6 (myeloma cells IL-2t6, a human lT cell leukemia line; kindly provided by Federica Sallusto, Institute for Research in Biomedicine, Bellinzona). The cell culture was cultivated in RPMI 1640 medium (Pan-Biotech, Aidenbach, Germany) supplemented with 2 mM glutamine (Pan-Biotech), 1% (vol/vol) non-essential amino acids (Gibco), 1% (vol/vol) sodium pyruvate (Gibco, Carlsbad, California, USA), 50 μg/ml penicillin-streptomycin (Corning, NY, USA), 0.00001% β-Mercaptoethanol (Gibco) and 5% inactivated human AB positive serum (Blood Bank Basel). Additional IL-2 was added every 4 days. Growing wells were transferred to 48/24 well plates and finally to 75 cm^3^ cell culture flasks until cells were fully rested (20–25 days). Cells were highly expanded in a single round of stimulation.

#### Peptide Stimulation

Peptides were synthesized by Peptides and Elephants GmbH (Henningsdorf, Germany) and dissolved in DMSO at a stock concentration of 5 mM. The peptides and their sequences which we used are listed in Supplementary Table 2. The response of PHA-expanded CSF-infiltrating CD4^+^ T cells to citrullinated or non-citrullinated myelin and the CEF (CMV, EBV, influenza virus, tetanus toxoid) (Peptides and Elephants GmbH), peptide pool was tested by seeding 6 × 10^4^ of expanded CSF-infiltrating CD4^+^ T cells and 2 × 10^5^ irradiated autologous PBMCs in quadruplicates per each condition of peptide stimulation or in the absence of peptides. Stimulation with anti-CD2/CD3/CD28 beads (Miltenyi) was used as additional positive control.

#### Proliferation Assay

The above described peptide stimulations of bulk CD4^+^ T cells from CSF of oligoclonal band (OCB) positive relapsing-remitting (RRMS) patients were then used to test T cell reactivity with autologous irradiated PBMCs as antigen-presenting cells. Proliferation of T cells was measured by ^3^H-thymidine incorporation. At day 2, the cells were pulsed with 1 μCi of methyl-^3^H-thymidine per well (Hartmann Analytic, Braunschweig, Germany) and harvested after 16 h onto a membrane (Filtermat A, GF/C, Perkin-Elmer, Waltham, Massachusetts, USA) using a semi-automated harvester (Tomtec, Hamden, Connecticut, USA). Incorporation was measured by β-scintillation counting (Wallac 1450, Perkin-Elmer). Proliferative responses were given as counts per min (cpm) and the stimulatory index (SI) was calculated as follows: SI = Mean (replicates cpm peptide)/Mean (replicates cpm without peptide).

#### Cytokine Measurement

After 48 h of incubation and before adding thymidine, 100 μl of cell culture supernatant were removed in order to test the cytokine secretion. Here, CD4^+^ T cell reactivity to peptides was analyzed in supernatants for IFN-γ using an IFN-γ ELISA (Biolegend, San Diego, California, USA) according to manufacturer's instructions. Cytokine production higher than 100 pg/ml was considered as a strong positive response.

### HLA Typing

Individuals were typed for HLA class I and -II alleles at Histogenetics LLC, NY, USA. Isolation of DNA from whole blood was performed with a standard DNA isolation protocol using a Triton lysis buffer and Proteinase K treatment. Purified genomic DNA with a final concentration of 15 ng/μl was used to type for HLA class I (A^*^ and B^*^) and HLA class II (DRB1^*^, DRB3^*^, DRB4^*^, DRB5^*^, DQA1^*^, and DQB1^*^) using high-resolution HLA sequence-based typing (SBT). The patients' information is summarized in Supplementary Table 3.

### Statistical Analysis

Pearson correlation analysis was performed between responses obtained from proliferation assay (thymidine incorporation) and cytokine secretion (IFN-γ) for MOG and CEF peptides.

## Results

### Characterization of Brain Tissue

To identify the different tissue types, e.g. normal appearing white matter (NAWM) or lesion tissue, sections from tissue blocks were immunohistochemically stained and analyzed (Figure 1A). Stainings were performed for MOG (A, D, G, J, M), and HLA-DR (LN3; B, E, H, K, N), as a marker for microglia and macrophages and luxol fast blue (C, F, I, L, O), as markers for myelin and oligodendrocytes (Figure 1A). Figure 1 shows representative staining for control- (C) and MS tissue. LFB and anti-MOG antibody staining identified sites of demyelination (Figures 1A:G,I). Staining with anti-HLA-DR antibody shows inflammatory cells (Figure 1A:H). Based on the staining, tissue types were defined and then cut for protein isolation. Tissue was taken from normal appearing gray matter (NAGM), normal appearing white matter (NAWM) as well as from active lesions from the diseased tissue. From control tissue, parts of gray (GMC)- and white matter (WMC) were excised. Figure 1B shows an immunohistochemistry staining of citrulline in a section of MS brain to illustrate the distribution of citrullination. We observed substantially higher citrullination staining in white compared to gray matter tissue.

### Identification of Specific Post-translationally Modified Peptides and Proteins in Pre-fractionated Brain Tissue Samples

**Figure 2:**
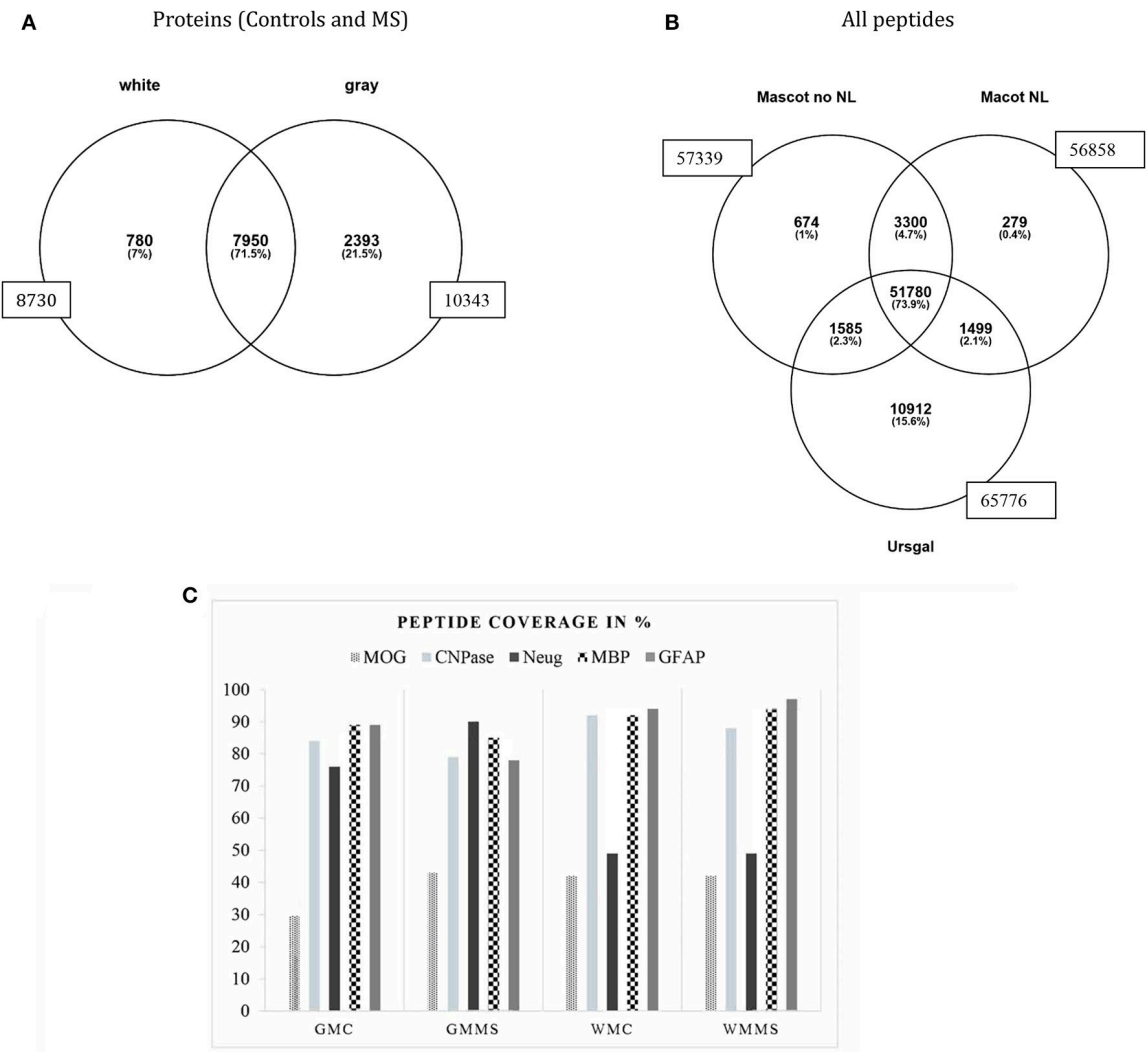
Overall distribution of proteins identified in white and gray matter (control and MS combined). A total number 10,343 proteins was identified in gray matter and 8,730 proteins in white matter with an overlap of 7,950 proteins in both tissues **(A)**. A number of 780 proteins were uniquely attributed to white matter and 2,393 proteins to gray matter. **(B)** Shows all peptide counts in the different search engines from white matter tissue (representative for all tissues analyzed). **(C)** This graph presents the percentage of tryptic peptide sequence coverage of the whole protein, MOG, CNP, Neurogranin, MBP, and GFAP and their identification in the four tissues analyzed, gray matter control (GMC), gray matter MS (GMMS), white matter control (WMC), and white matter MS (WMMS).

For proteomic analysis we decided to extract proteins from the brain tissue by barocycler. This technique allows to extract proteins efficiently from small sample sizes. It has the advantage of using a single test tube from tissue disruption until the tryptic digestion, thereby reducing the introduction of technical variations in the samples (Figure 1C). To be able to achieve a higher resolution of the brain proteome we decided to render the samples less complex and therefore easier to analyze. We first separated our peptide digests by hydrophilic interaction chromatography (HILIC), and then two fractions were pooled and injected into liquid chromatography coupled to a mass spectrometer (see illustration in Figure S2). The pre-fractionation process allowed us to identify 10,343 proteins in gray matter and 8,730 proteins in white matter tissue. These numbers correspond to combined data from control and MS patients (Figure 2). The higher number of proteins in gray matter tissue is not surprising since gray matter tissue is more densely packed with cells than white matter tissue. Overall, 7,950 proteins were common in both groups, white and gray matter. In gray matter 2,393 proteins could be uniquely identified compared with 780 in white matter (Figure 2A). Since we were interested in the fraction of proteins involved in processes of inflammation and the immune system an analysis with “STRING-DB” (https://string-db.org/) was performed. We submitted the entire protein list that we had obtained from MS white matter and control tissue. The MS white matter showed a network of proteins involved in immune reactions, which were completely absent in WM control. Control tissue showed an enrichment of a protein network of the nervous system (data not shown).

### Identification of Myelin Sheath- and Other CNS Proteins

An important first step for the comparison of different brain tissue samples is to assure that the protein extraction steps worked with equal efficiency for the different samples. For that reason, we analyzed “representative proteins” from the myelin sheath and considered their presence as quality control for the extraction efficiency. For myelin proteins, myelin basic protein (MBP), myelin oligodendrocyte protein (MOG), myelin-associated glycoprotein (MAG), myelin proteolipid protein (PLP), and 2′,3′-cyclic nucleotide 3′-phosphodiesterase (CNPase), for astrocytes, glial fibrillary acidic protein (GFAP), and neurogranin as neuronal marker, we identified a high number of peptides covering a major part of the respective protein sequences. Figure 2C shows the peptide coverage (in %) of these proteins. For MBP the coverage is optimal with more than 90%. The sequence coverage of MOG was between 30 and 40% and for CNPase around 80%. Peptide sequence coverage of GFAP was more than 90% and for neurogranin more than 45%. These numbers indicate a comparable efficiency of peptide extraction from the tissues analyzed.

### Identification of “*in vivo*” Citrullinated Proteins

Our main goal was to analyze as detailed as possible, which proteins are citrullinated in MS- and control brain tissue and also for the main regions of the brain, i.e., gray and white matter. In Figure 2B, we show the identification of all peptides from white matter from control and MS. The search with “Mascot no NL” (without neutral loss) identified 57,339 peptides, “MascotNL” (Mascot, with neutral loss) revealed 56,858 peptides and “Ursgal” identified 65,776 peptides. The majority of peptides was common to all three algorithms; 51,780 peptides. Unique to the individual search engines were 674 peptides for “Mascot no NL,” 279 to “MascotNL” and 10,912 peptides for “Ursgal” (Figure 2B). Similar distributions were observed for gray matter tissue (data not shown).

**Figure 3:**
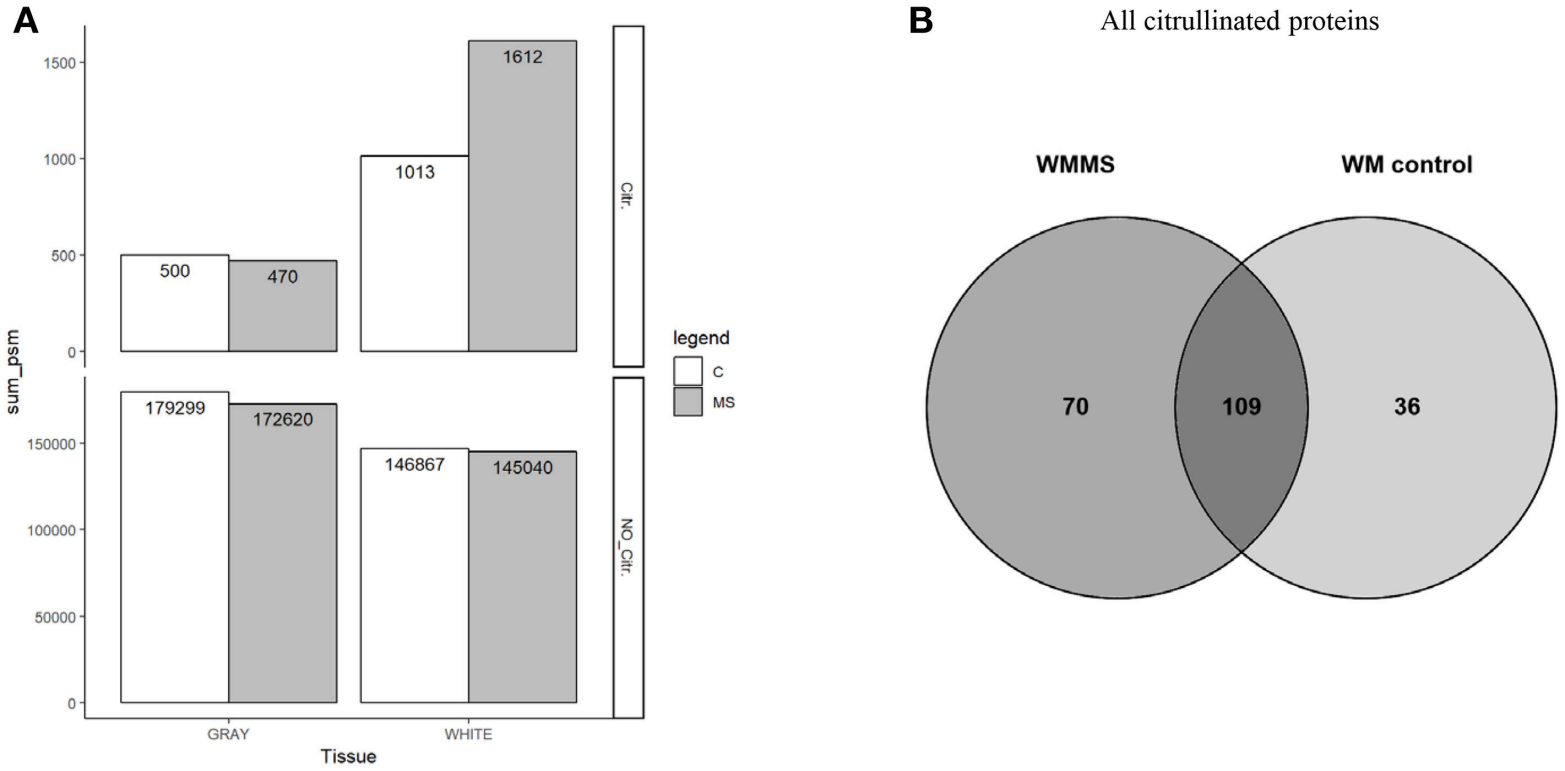
**(A)** The number of spectrum matched peptide of citrullinated peptides in all tissues are shown in upper panel (Citr.). All peptide spectrum matches found in all four types of tissue gray (GM) and white matter are shown (WM) in lower panel (NO_Citr.). **(B)** Number of citrullinated proteins that were identified in white matter tissue. From the total amount of 179 proteins in MS tissue and 145 in controls 70 proteins were unique in MS tissue and 36 proteins in control tissue with an overlap of 109 proteins in both tissues.

We based the further analysis on combined identification from the software searches in Mascot (no NL), Mascot (NL) and “Ursgal.” For most of the following analysis, we used peptides, which had been identified by at least 2 of the search engines since not all of them could be identified with all three. The distribution of all spectral peptide matches and citrullinated spectral peptide matches and their corresponding proteins numbers from the different tissues are illustrated in Figure 3 and Supplementary Data 1. The highest number of citrullinated spectral matches peptides was found in WM tissue (Figure 3A). The lower part of the panel labeled with NO_Citr., represents the overall number of spectral matches from the four different tissues and shows a higher number in GM tissue. Further, very similar numbers were found for gray matter of controls and MS (179,299 and 172,620), and similarly also for white matter tissue (146,867 in controls and 145,040 in MS). In Figure 3A (Citr., upper part of the panel) the peptide spectral matches show a strong increase of the citrullinated fraction in the white matter tissue of MS (1,612) vs. control (1,013). In contrast, in gray matter tissue, the numbers were slightly lower in MS tissue (470) compared to control (500).

**Figure 4:**
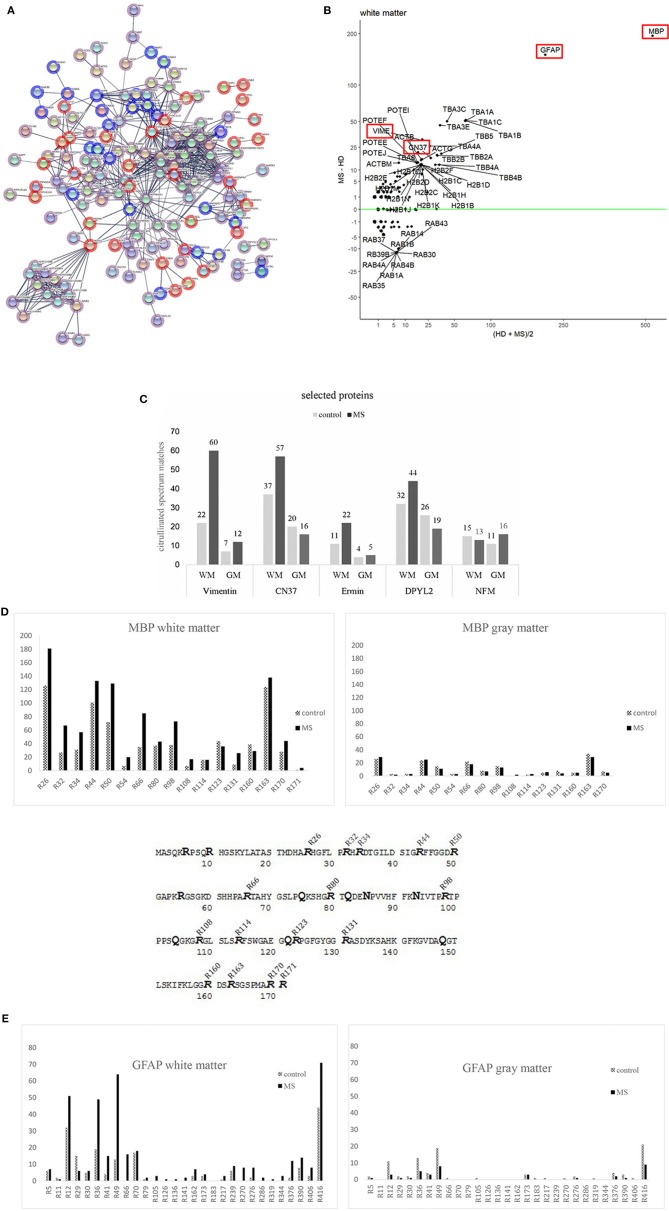
**(A)** A StringDB representation of citrullinated proteins in MS (red) and controls (blue) tissue. Edge settings is high-confidence (minimum required interaction score 0.700). Overlapping in both tissues are depicted in pink. **(B)** Altman Bland plot. Vertical axis shows the mean number of peptide spectrum matches against citrullinated sequences in controls and MS donor for white matter. The vertical axis shows the differences in PSM between MS and controls. Highlighted in Red are proteins discussed in the text. **(C)** This figure shows a list of other most citrullinated proteins. Overall numbers of tissue distributions are shown: Vimentin, 2′,3′-cyclic-nucleotide 3′-phosphodiesterase (CN37), ermin (juxtanodin), dihydropyrimidinase-related protein 2 (DPYL2 or crmp2) and neurofilament medium polypeptide (NFM). Most of the proteins show a higher range of citrullinated peptides in white matter of diseased tissue. Only NFM is citrullinated in a similar manner in all tissues analyzed. **(D)** Distribution of citrullinated sites in one of the major citrullinated proteins in brain tissue MBP. The bars represent the numbers of modified arginine sites in corresponding tissue of control and MS cases. Peptide spectral matches, which were used for quantification of the modified sites are shown as supplements. MBP sequence (P02686-5) with modified arginine found in our analysis. 16 out of 19 arginine sites were found to be citrullinated in control and diseased brain (MS) tissue. Sites R6, R10, and R55 were never found to be altered. We never found R171 to be citrullinated in gray matter tissue. **(E)** Distribution of citrullinated sites in another major citrullinated protein in brain tissue GFAP. The bars represent the numbers of modified arginine sites in corresponding tissue of control and MS cases. Peptide spectral matches, which were used for quantification of the modified sites are shown as supplements.

With respect to overall peptide numbers, there is little difference between MS and control tissue (lower panel). The elevated numbers of citrullinated peptides in white matter therefore indicate a specific enrichment of modified peptides. The diagram in Figure 3B shows the number of citrullinated proteins. Here we only show white matter proteins in control and MS. From the 179 proteins in MS white matter (WMMS) and 145 in control white matter (WMC), we found 36 citrullinated proteins to be unique to white matter tissue and 70 unique to gray matter with an overlap of 109 in both tissues. A more detailed analysis of these citrullinated proteins (>80) is shown in Table 1 as a non-exhaustive list. It represents a selection of citrullinated proteins, for which we identified at least two citrullinated peptides. We grouped the proteins into functional or cellular processes, such as myelin-associated proteins, neuronal development, neuronal skeleton, astrocytes, synapse, energy transduction, membrane trafficking, cytoskeleton in general immune response and anti-apoptotic activity. The distribution and the possible interactions of the citrullinated proteins are illustrated in Figure 4A in the protein interaction network generated by String (24). Figure 4B shows the differences regarding citrullination as numbers of peptide-spectrum matches between MS and controls. For GFAP and MBP much higher numbers of spectra matching citrullinated sequences were detected in MS than in controls. Other proteins worth mentioning are vimentin (VIME) and CNPase (CN37). Proteins with strong citrullination pattern, i.e. more than three citrullination sites, are represented in Figure 4C.

**Table 1:** List of proteins with at least two citrullinated peptides identified in classified in functional groups.

**Myelin associated proteins**	**Synapse**
P02686-5 MBP_HUMAN Myelin basic protein P20916|MAG_HUMAN Myelin-associated glycoprotein P09543|CN37_HUMAN 2′,3′-cyclic-nucleotide 3′-phosphodiesterase Q92597|NDRG1_HUMAN Protein NDRG1 Q8TAM6|ERMIN_HUMAN Ermin Q13875|MOBP_HUMAN Myelin-associated oligodendrocyte basic protein	Q92686|NEUG_HUMAN Neurogranin P61764|STXB1_HUMAN Syntaxin-binding protein 1 Q8N3V7|SYNPO_HUMAN Synaptopodin Q9C0H9|SRCN1_HUMAN SRC kinase signaling inhibitor 1 Q13424|SNTA1_HUMAN Alpha-1-syntrophin
**Neuronal development**	**Membrane trafficking**
Q09666|AHNK_HUMAN Neuroblast differentiation-associated protein AHNAK O15075|DCLK1_HUMAN Serine/threonine-protein kinase DCLK1 P78324|SHPS1_HUMAN Tyrosine-protein phosphatase non-receptor type substrate 1 Q16555|DPYL2_HUMAN Dihydropyrimidinase-related protein 2 Q14195|DPYL3_HUMAN Dihydropyrimidinase-related protein 3P21291|CSRP1_HUMAN Cysteine and glycine-rich protein 1	Q9NRW1|RAB6B_HUMAN Ras-related protein rab6B P63027|VAMP2_HUMAN Vesicle-associated membrane protein 2 **Chaperonine like activity** P02511|CRYAB_HUMAN Alpha-crystallin B chain P07900|HS90A_HUMAN Heat shock protein HSP 90-alpha O95817|BAG3_HUMAN BAG family molecular chaperone regulator 3
**Neuronal skeleton**	**RNA binding proteins**
P10636|TAU_HUMAN Microtubule-associated protein tau P07196|NFL_HUMAN Neurofilament light polypeptide P12036|NFH_HUMAN Neurofilament heavy polypeptide Q16352|AINX_HUMAN Alpha-internexin **Astrocyte specific** P14136|GFAP_HUMAN Glial fibrillary acidic protein	P61978|HNRPK_HUMAN Heterogeneous nuclear ribonucleoprotein K P22626|ROA2_HUMAN Heterogeneous nuclear ribonucleoproteins A2/B1 P38159|AUXI_HUMAN RNA-binding motif protein, X chromosome P23588|IF4B_HUMAN Q14011|CIRBP_HUMAN Cold-inducible RNA-binding protein P68104|EF1A1_HUMAN Elongation factor 1-alpha 1 P38159|RBMX_HUMAN RNA-binding motif protein, X chromosome
**Membrane signaling**	**Histone**
Q8N7J2|AMER2_HUMAN APC membrane recruitment protein 2 Q9NZH0|GPC5B_HUMAN G-protein coupled receptor family C group 5 member B	P62807|H2B1C_HUMAN Histone H2B type 1-C/E/F/G/I
**Cytoskeleton**	**Cell adhesion**
Q13885|TBB2A_HUMAN Tubulin beta-2A chain Q9UEY8|ADDG_HUMAN Gamma-adducin P04350|TBB4A_HUMAN Tubulin beta-4A chain P07437|TBB5_HUMAN Tubulin beta chain Q9BQE3|TBA1C_HUMAN Tubulin alpha Q71U36|TBA1A_HUMAN Tubulin alpha-1A P46821|MAP1B_HUMAN Microtubule-associated protein P11137|MTAP2_HUMAN Microtubule-associated protein P60709|ACTB_HUMAN Actin, cytoplasmic 1 O94811|TPPP_HUMAN Tubulin polymerization-promoting protein Q9BW30|TPPP3_HUMAN Tubulin polymerization-promoting protein family member 3 P35611|ADDA_HUMAN Alpha-adducin Q8N7J2|AMER2_HUMAN APC membrane recruitment protein 2 Q14847|LASP1_HUMAN LIM and SH3 domain protein 1 P06396|GELS_HUMAN Gelsolin O01082|SPTB2_HUMAN Spectrin beta chain, non-erythrocytic 1 Q96PY5|FMNL2_HUMAN Formin-like protein 2 O43491|E41L2_HUMAN Band 4.1-like protein 2 O75122|CLAP2_HUMAN CLIP-associating protein 2 O75781|PALM_HUMAN Paralemmin-1 Q13813|SPTN1_HUMAN Spectrin alpha chain, non-erythrocytic 1 Q92614|MY18A_HUMAN Unconventional myosin-XVIIIa Q16181|SEPT7_HUMAN Septin Q15149|PLEC_HUMAN Plectin Q14244|MAP7_HUMAN Ensconsin Q9H3Q1|BORG4_HUMAN Cdc42 effector protein 4 Q765P7|MTSSL_HUMAN MTSS1-likEprotein P35241|RADI_HUMAN Radixin Q9H9H5|MA6D1_HUMAN MAP6 domain-containing protein 1 **Nuclear membrane** P20700|LMNB1_HUMAN Lamin-B1 P02545|LMNA_HUMAN Prelamin-A/C Q9H910|HN1L_HUMAN Hematological and neurological expressed 1-like protein	Q07157|ZO1_HUMAN Tight junction protein ZO-1 Q9UDY2|ZO2_HUMAN Tight junction protein ZO-2 P26232|CTNA2_HUMAN Catenin alpha-2 **Cell matrix interaction** Q14CZ8|HECAM_HUMAN Hepatocyte cell adhesion molecule P78333|GPC5_HUMAN Glypican-5 **Endocytosis** O75061|AUXI_HUMAN Putative tyrosine-protein phosphatase auxilin O00193|SMAP_HUMAN Small acidic protein Q9UBC2|EP15R_HUMAN Epidermal growth factor receptor substrate 15-like 1 **Phosphatase inhibitor** Q96A00|PP14A_HUMAN Protein phosphatase 1 regulatory subunit 14A **Endoplasmic Reticulum** Q9UNZ2|NSF1C_HUMAN NSFL1 cofactor p47 **Energy transduction** P12277|KCRB_HUMAN Creatine kinase B-type P11216|PYGB_HUMAN Glycogen phosphorylase, brain form **Immune response** P43243|MATR3_HUMAN Matrin-3 P17858|PFKAL_HUMAN ATP-dependent 6-phosphofructokinase

Vimentin, an intermediate filament protein, is important for maintaining the structure of a cell. It had already been described in AD (25) and is one of the most citrullinated proteins besides MBP and GFAP in WMMS. We found an increased number of modifications in both tissues of MS in comparison to controls.

CNPase is an oligodendrocyte-specific protein and one of the most abundant proteins in CNS myelin. Its function remains unknown. CN37 has been described as an autoantigen in MS (26) and had been identified to be citrullinated in a mouse model (27). We identified a higher number of citrullinated CN37 peptides in human brain white matter tissue as compared to published data (28). In gray matter, we noticed slightly more citrullinated spectral matches in controls.

We describe for the first time citrullinated sites of members of neurofilament protein family (NFL, NFM and NFH), DPYL2 (dihydropyrimidase-related protein 2, CRMP2), a protein involved in neuronal development and polarity (29), and human ermin, a protein playing a role in myelin development and maintenance and stability of myelin sheath (30). Among the three members of neurofilament proteins, there was no difference with regard to citrullination across tissues and donors. We show neurofilament medium chain (NFM) as a representative member of that group.

The protein DPYL2 showed a particularly increased number of modified peptides in WMMS tissue and a slight increase in GMC.

The protein with the highest number of modified/citrullinated peptides in all tissues was MBP followed by GFAP, which will be described in more detail. The analysis of total spectral counts showed that overall comparable amounts of spectra were observed in control and MS tissue of gray matter, and in white matter citrullinated spectral counts were strongly increased in MS white matter whereas the number of citrullinated spectral counts in gray matter was slightly reduced in MS tissue.

### Citrullination and Other Modifications of Myelin Basic Protein

We examined in more detail MBP as one of the import proteins of the myelin sheath and due to its role as target in autoimmune responses. MBP can undergo post-translational modifications at various sites. Those modifications are phosphorylation, methylation, oxidation, citrullination and deamidation/isomerization. So far, very little is known about the implication of these modifications in disease processes in MS (31–33). In our study, we found 16 out of the 19 arginine sites to be modified into citrulline (Figure 4D). Earlier studies described 6–9 arginine sites in MBP to be citrullinated in MS “*in vivo*” (32, 34). In these studies, MBP was purified prior to analysis, while we analyzed whole tissue extracts from histologically characterized sites. We observed various modifications on different amino acids and will describe some of them in more detail. The peptide MBP (77–92) “(K)SHG**R**T**Q**DE**N**PVVHFFK(N)” was modified at multiple sites. Modifications occurred at asparagine (N-85), arginine (R-80), and glutamine (Q-82); DENP, HGRT, and RTQD. The peptide “(K)GVDA**Q**GTLS(K)” (144–154) was deamidated at DAQG (Q-148). We mention these deamidations since these modifications have been analyzed previously in MS patients and healthy donors (32, 35). These two reports show that deamidation of the latter peptide (MBP 144–154) can increase with age in MS patients and others in animals (32). The deamidation of glutamine (Q) in the peptide sequence (82–90) (QDENP) has been shown to block its degradation by the protease cathepsin-D in Alzheimer's Disease (36). In our analysis, we found that 15 (16) identified citrullination sites were present in control as well as in MS tissue, in gray—and in white matter. A 16th position could only be found in WMMS.

To see if there is any specific citrullination pattern of MBP, we counted the number of peptides and the respective citrullinated sites and plotted them over the whole protein sequence (Figure 4D). Citrullination is not unique to MS tissue and neither in GM nor WM tissue. Some citrullinated sites were strongly over-represented in MS tissue, i.e., R26, R32, R34, R44, R50, R66, and R98. In GM, only the sites R26 and R44 were slightly more citrullinated in MS tissue compared to control.

The situation for the structural protein GFAP looked similar. GFAP protein was found to be much more modified in WM as compared to GM from MS tissue. Highly modified sites among others were positions R12, R36, R41, R49, and R390 and R416. Similar to MBP, GM tissue generally shows a much lower state of citrullination and a relatively higher rate in control tissue (Figure 4E; Supplementary Data 1). Since citrullination depends on the activity of PADI, we looked for the presence of PADI in the tissues. We could identify a substantial number of peptides from the isoform PADI2 (between 18 and 20 peptides across all tissue representing between 32 and 45% of protein sequence, data not shown). No peptide of PADI4, the other isoform described to be present in CNS (17), could be found.

### Immunological Reactivity Against Citrullinated MBP Peptides

**Figure 5:**
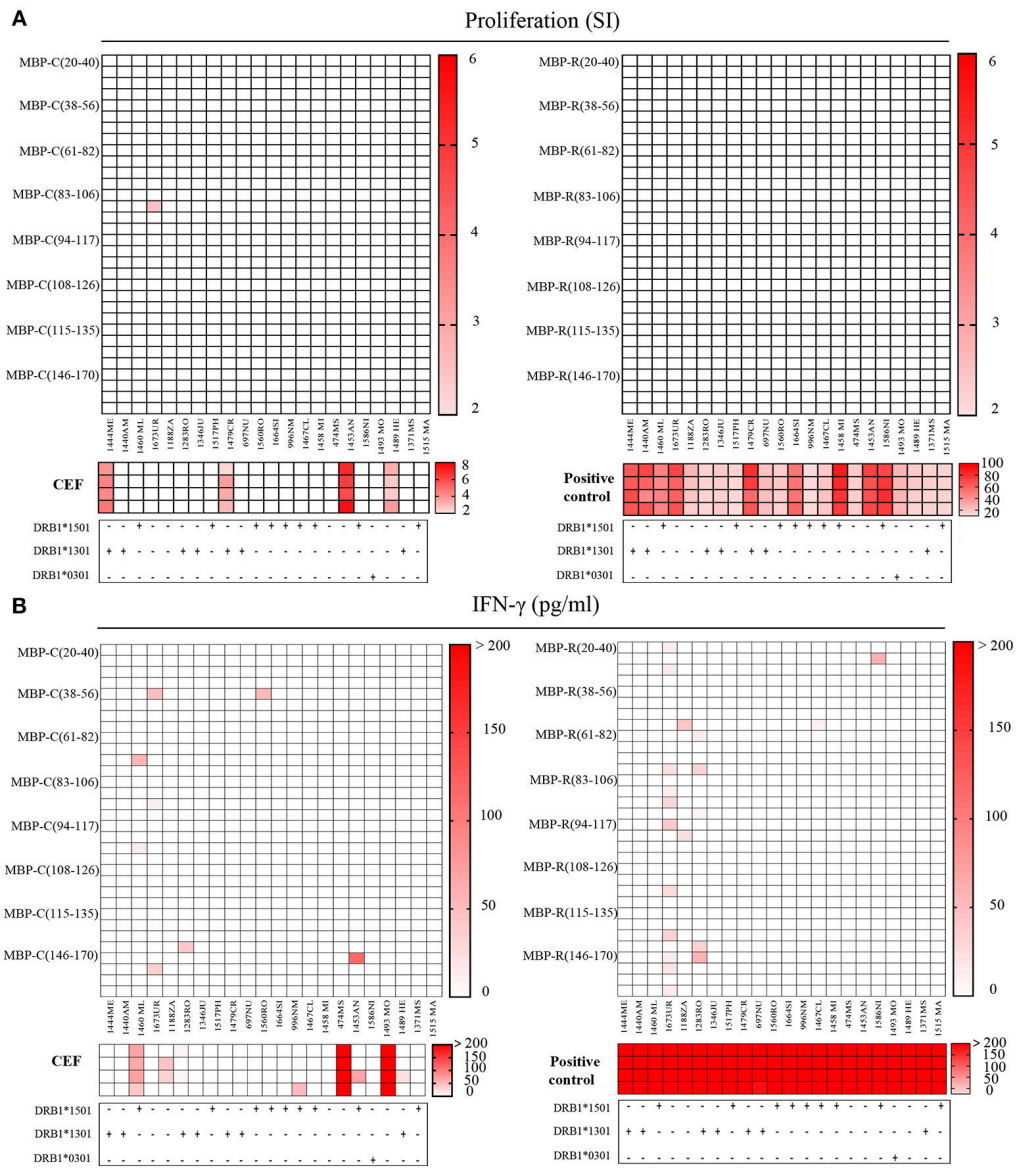
Recognition of citrullinated vs. non-citrullinated MBP in CSF-infiltrating T helper cells of MS patients. **(A,B)** Reactivity of CSF-infiltrating CD4^+^ T cells from untreated MS patients to citrullinated MBP (left) and non-citrullinated MBP peptides (right), using irradiated autologous PBMCs as antigen-presenting cells. Each square represents one well. MS risk-associated HLA-DRB1 alleles are reported for each individual under the respective graph. **(A)** Proliferative responses to MBP peptides, CEF peptides or anti-CD2/CD3/CD28 stimulation as positive control are given as stimulatory index (SI). The strength of the response is depicted by color coding. A SI > 2 is considered as positive response. **(B)** Responses detected by IFN-γ secretion against to MBP peptides or CEF peptides in the same wells that have been tested in the proliferation assay. The IFN-γ concentration in culture supernatants is depicted as pg/ml.

In order to find out if the citrullinated MBP peptides are targeted by the immune system we examined CSF-infiltrating CD4^+^ T cells from 22 MS patients. We analyzed CSF-derived T cells under the assumption that they are more likely to be biologically relevant in MS than peripheral blood lymphocytes, since the T cells have already infiltrated the CNS compartment. For that purpose, CD4 T cells were freshly isolated and expanded as described (16) from CSF of 19 relapsing remitting MS, 2 primary progressive MS and 1 clinically isolated syndrome patients (Table 2 and Supplementary Table 3), and, subsequently tested in quadruplicates for eight newly identified citrullinated MBP peptides together with the non-modified peptides. Furthermore, seven immunodominant myelin peptides were examined, and, only for MBP, the most abundant citrullinated epitopes were included in the assay (Supplementary Table 2). Proliferation and IFN-γ production were used as functional readouts. Positive responses to CEF, a peptide pool of CMV, EBV, influenza virus and tetanus toxoid, and global T cell stimulation by anti-CD2/CD3/CD28 beads were tested in parallel as positive controls. Four patients (1444ME, 1479CR, 1453AN, 1489HE) showed responses to CEF peptides with a stimulation index (SI) > 2, but we did not observe specific recognition of citrullinated- and non-citrullinated MBP peptides in proliferation assays (Figure 5A). When we analyzed the IFN-γ secretion in the culture supernatant of the same wells tested for proliferation, we did also not find strong IFN-γ release upon exposure to citrullinated MBP peptides, but weak responses in only a few patients and one of the four replicate wells (Figure 5B). On the other hand, in two patients (1673UR, 1283RO), IFN-γ secretion (~50 pg/ml) in response to several non-modified epitopes of the MBP protein was seen (Figure 5B). Given these results, we conclude that the non-modified peptides are more frequently recognized by CSF-infiltrating CD4^+^ T cells compared to the citrullinated version and that the reactivity to the latter is overall very low.

**Table 2:** Main clinical information and CSF findings of MS patients.

	**MS patients (22 subjects)**
Age (y)	37.3 ± 12.6 (17–58)
Gender (F/M)	15/7
Disease duration (y)	1.6 ± 4.4
Patients with disease duration < 12 months (%)	16 (73%)
Time from last relapse (m)	1.3 ± 2.2 (0–8)
Patients with MRI-LP delay < 1 month (%)	20 (91%)
MRI active patients (%)	11 (50%)
CSF-restricted IgGOCB	21 (95.5%)
Patients with IgG Index > 0.70 (%)	15 (68.2%)
Blood-brain barrier damage (%)	5 (22.7%)
CSF cell count (/μL)	7 ± 3

*Disease duration was defined as the time-span between disease onset and lumbar puncture (median value and range are reported). The “time from last relapse” value did not include Primary Progressive MS data. As indicated by the narrow interval between MRI and LP (MRI-LP delay), the great majority of MS patients received MRI the day before LP. Blood Brain Barrier (BBB) damage was considered when the albumin quotient (Q_Alb_) exceeded the normal value for patient's age (i.e., age/15 + 4). y, years; m, months*.

Global T cell stimulation resulted in clear responses in all donors, whereas responses to CEF peptides were less frequent. The reactivity observed in proliferation assays to CEF positive control peptides was partially paralleled by IFN-γ release. Only two out of the four patients (1453AN, 1489HE) responding in thymidine incorporation assay, produced also IFN-γ at high concentrations (~400 pg/ml). However, additional patients (1460ML, 1188ZA) responded to these antigens (Figure 5B).

**Figure 6:**
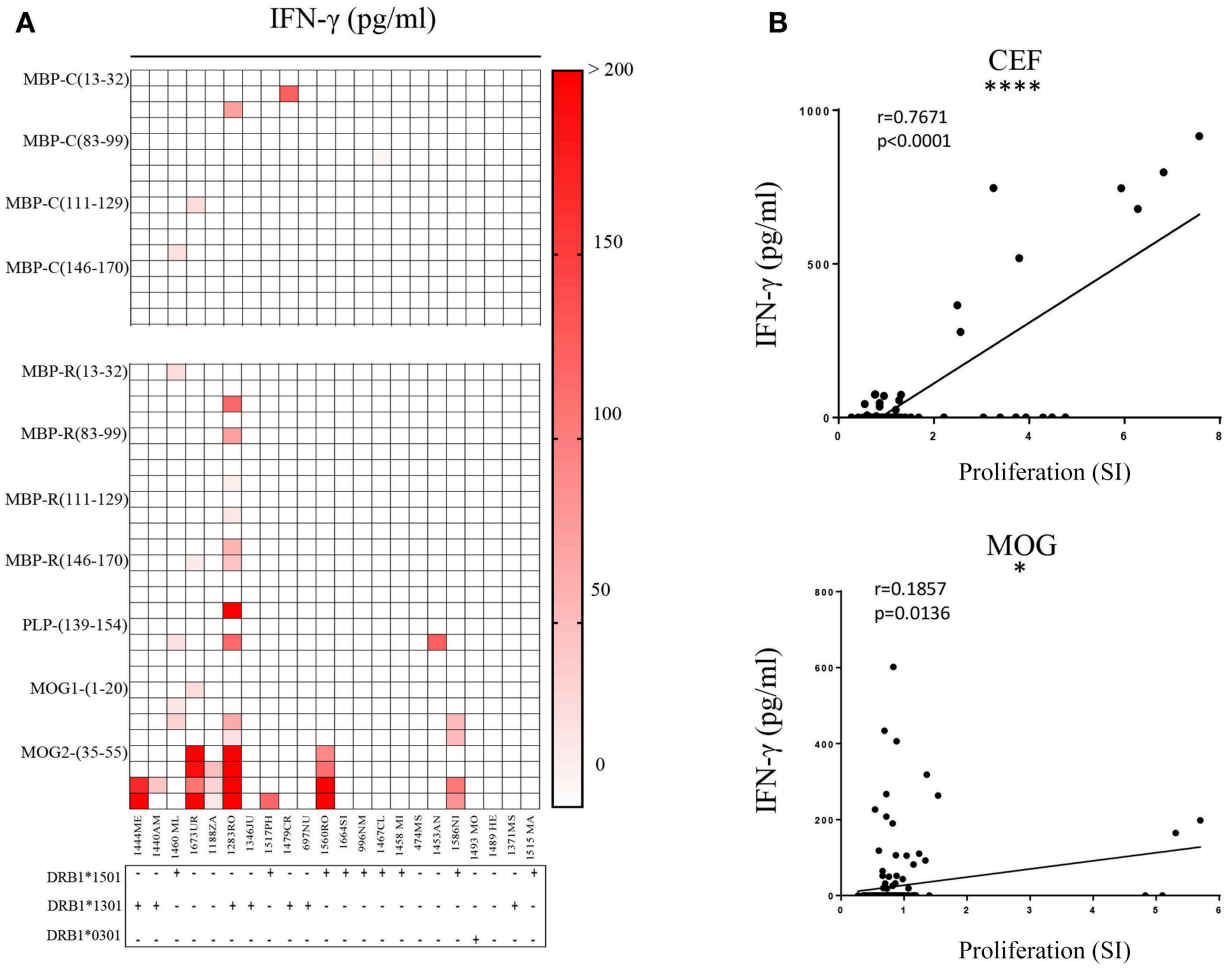
Recognition of citrullinated vs. non-citrullinated immunodominant myelin peptides and correlation of readouts. **(A)** Reactivity of CSF-infiltrating CD4^+^ T cells to immunodominant MBP citrullinated (up) and immunodominant myelin (down) peptides, using irradiated autologous PBMCs as antigen-presenting cells. Each square represents one well. MS risk-associated HLA-DRB1 alleles are reported for each individual under the respective graph. Responses detected by IFN-γ secretion in culture supernatants are depicted as pg/ml. **(B)** Pearson correlation analysis is performed for MOG and CEF peptides. Data obtained from ELISA and proliferation assay are compared and each dot represents one well-tested. Significant strong correlation between the readouts is detected for CEF (*p* < 0.0001) while for MOG a mild correlation is shown (*p* = 0.0136).

When examining other immunodominant myelin peptides (9, 37) we observed in most cases IFN-γ secretion. Several patients reacted clearly to MOG2 (35–55) peptide (Figure 6A). 1444ME showed proliferation with a stimulatory index (SI) of 5 (data not shown), 1673UR, 1283RO, and 1560RO release of IFN-γ (~300 pg/ml). 1283RO responded with high IFN-γ release to other non-citrullinated, immunodominant MBP peptides (Figure 6A) in comparison to the citrullinated version (Figure 6A). These data show that bulk CSF-infiltrating CD4^+^ T cells of MS patients are able to recognize at the same time different epitopes of the same protein but also different antigens. No significant association between peptide recognition and the MS risk-associated HLA-DRB1 alleles, i.e., DRB1^*^15:01, DRB1^*^13:01, and DRB1^*^03:01, was observed (reported below the graphs).

Since we observed substantial differences in the results obtained from the two response readouts, i.e., thymidine incorporation vs. IFN-γ release, we wanted to assess a correlation between the two measures and applied Pearson Correlation testing on CEF- and MOG peptides responses. We observed (Figure 6B) a strong positive correlation (*r* = 0.7671) for CEF peptides (*p* < 0.0001), but only a weak correlation for MOG (*r* = 0.1857), where the majority of responses were detected by ELISA. These results show that IFN-γ secretion appeared to be more sensitive than proliferation as readout.

## Discussion

We investigated the composition of citrullinated proteins from human post-mortem brain tissue. Tissues were characterized by immunohistochemistry staining with antibodies against MOG and HLA-DR and LFB. These markers allowed to distinguish tissue with lesions from NAWM. Some of the tissue sections showed increased staining for citrullination in white matter compared to gray matter tissue, an observation that correlated well with our proteomic findings. Based on mass-spectrometry, we could identify a high number of citrullinated proteins, which far exceeded the numbers already published in human CNS (9, 38). By establishing a “spectral peptide library” from different disease-relevant brain tissues and controls, we provide a basis for further, more extensive investigation of the MS brain proteome. We combined an optimized protein extraction technique based on PCT (pressure cycle technology) with chromatographic pre-fractionation, HILIC, to obtain high proteome coverage. We searched our mass spectrometry data with various search engines to distinguish post-translational modifications and identify citrullination. The difficulty of correctly identifying minute mass changes, i.e., an increase of 1 Da per citrullinated site, made it necessary to apply complementary bioinformatics approaches to validate the results, since another post-translational modification, deamidation, which also increases the molecular mass by 1 Da, occurs in aging tissue on asparagine (N) and glutamine (Q) and can be a source of misinterpretation. Therefore, we used different algorithms to interpret the spectra and features, which are inherent of citrullinated proteins, i.e., resistance to tryptic digestion and the neutral loss of 43 Da inside the mass spectrometer instrument (39). Most of the citrullinated proteins we identified, as well as new citrullinated sites of MBP had not been described in MS tissue before. So far, citrullinated myelin proteins had been analyzed from excised bands (SDS-PAGE) or after “*in vitro*” citrullination, but not from entire tissue. We used two software “Mascot” and “Ursgal” to identify citrullination. Depending on the search parameters and software the number of identified peptides varied as illustrated in Figure 2B. We could not detect any of the N-terminal arginines to be citrullinated. This phenomenon had been reported earlier concluding that “N-termini” are less prone to be citrullinated (38).

Citrullination facilitates enzymatic degradation of MBP but in the situation of increased citrullination, especially of myelin proteins, scavenger cells like macrophages might have difficulties coping with a high amount of proteins to degrade. It could also be that the presence/absence or activity of specific proteases like cathepsins play a role.

Citrullinated residues can be considered “neo-antigens” since they are not necessarily available during thymic selection of T cells and since citrulline is not one of the naturally occurring L-amino acids. Hence, T cells with high avidity T cell receptors against citrullinated peptides, which are presented via MHCII molecules, might escape negative selection in the thymus and target citrullinated peptides in the CNS. Earlier data from testing PBMCs and PBMC-derived T cell lines with proteolytic fragments (13) and 6 modified arginines in MBP, which had been known at that time (40), had indicated increased reactivity against citrullinated MBP epitopes. However, these data were preliminary due to incomplete knowledge of the citrullinated sites and other limitations. After analyzing in detail the possible citrullination sites in the present study, we wanted to expand the prior data by testing bulk CSF-infiltrating CD4^+^ T cells, i.e., from the CNS compartment, against MBP peptides containing the newly identified citrullinated sites and against control antigens. These studies aimed at the question if T cell reactivity against citrullinated epitopes of MBP is increased in MS as it has been described in a subset of rheumatoid arthritis patient for antibody reactivity against citrullinated peptides (41), and, if not generally increased in MS, whether it is found in a subset of patients. Our findings show that there is very little reactivity again citrullinated MBP epitopes and that it is thus unlikely to play a role in the autoimmune response in MS. When comparing the present study with previous data (12, 13), the testing of CSF-infiltrating T cells, which are more likely to be disease-relevant than PBMC-derived T cells, and of a larger number of individuals are the most important differences. The fact that we observed reactivity against MOG- and CEF peptides in a number of individuals, indicate that the lack of reactivity against citrullinated MBP peptides was not a technical problem. The observation that some wells were only positive when testing for IFN-γ, is likely explained by the fact that individual functions of T cells require different strengths of stimulation (42). Modified/citrullinated peptides may be less potent ligands compared to native peptides and, since only one antigen concentration was tested, it is possible that responsiveness was only observed for one functional readout instead of both (42).

Since we did not examine antibody reactivity against citrullinated proteins, the possibility remains that humoral responses could still play a role in MS as is the case in RA (42). Bodil et al. did not find elevated levels of antibodies either against citrullinated proteins or PAD in MS patients (43). Furthermore, decreased reactivity against citrullinated MBP was found in serum and CSF of MS patients. However, this study examined only two citrullinated MBP peptides (44).

Besides myelin proteins, we also identified additional citrullinated sites in vimentin and CN37 from WMMS, whereas numbers of CN37 and DPYL2 citrullinated peptides were slightly increased in GMC tissue (Figure 4C). Some of the newly identified citrullinated proteins like ermin (juxtanodin) and DPYL2 (crmp-2) are of particular interest. Little is known about ermin. It appears to be expressed only by oligodendrocytes and involved in the compaction of myelin (45) and the formation of axonal microtubules (46). It is interesting to note that citrullinated ermin is present in tissue were compaction of myelin is lost. Moscarello et al. were the first to hypothesize that damage of white matter in MS results from a failure to maintain compact myelin sheaths due to an increased citrullination of MBP (47). Citrullination of ermin may occur as “collateral damage.” MBP like ermin belongs to a group of proteins, which are characterized as “intrinsically disordered proteins” that adopt tertiary structure depending on the molecular environment (48). DPYL2 or (CRMP2) is a protein, which has been linked to neurodegenerative disorders (49) and shown to be involved in synaptic function. It appears in an interactome with proteins involved in B cell differentiation (49) and in T cells in the context of neuroinflammation in an animal model (50). Further, it has been shown to have multiple PTMs and potentially many interactors, among them structural proteins as tubulin (51).

Our list of citrullinated proteins shows a large number of molecules involved in cytoskeleton formation, especially vimentin, GFAP, tubulin and actin. The picture we obtained from our MS tissues indicates that structural proteins are the main targets of this particular post-translational modification. Citrullination is considered to result from insult and damage, leading to molecular and cellular breakdown. In addition, recent publications show that post-translational modifications as citrullination and deamidation also occur in the aging brain (32, 34, 36). Therefore, it is possible that the citrullination patterns we obtained from control brain reflect the natural aging of brain tissue. Nevertheless, citrullination occurred with a much higher frequency in MS tissue as compared to control. This could be due to inflammation, even if tissue is not overtly inflamed and considered “normal-appearing,” and support the argument that citrullination is not an initiator of the disease but the result. Other PTM, such as phosphorylation occur also on MBP, but their possible influence on disease course is currently not clear.

In summary, our study provides a comprehensive analysis of citrullinated peptides in white- and gray matter of MS patients and controls. We combined efficient protein extraction- and separation techniques to analyze very small samples. Thorough data mining with the support of complementary software allowed us to establish a map of citrullinated peptides and proteins. This proteomic approach in principle provides the basis for multiple other studies on the role of citrullination in MS brain tissue, but more broadly also with respect to analyzing other post-translational modifications in small tissue samples and identifying potential neo-antigens. This information, i.e., whether structural proteins and or those involved in inflammatory processes are citrullinated, should improve the understanding whether citrullination is implicated in distinct pathomechanisms in MS. Altered myelin, either via structural alterations, during the processes of de- and remyelination, neuronal/axonal loss or autoimmune inflammation could result in neoantigens and thereby induce an autoimmune reaction or increase demyelination (5).

The immunological testing in the present study focussed on citrullinated MBP epitopes based on previous reports, and, even though we examined CSF-infiltrating CD4^+^ T cells, we did not find a marked response, which argues against a major pathogenetic involvement of autoimmune T cells directed against citrullinated MBP epitopes in MS.

## Author Contributions

WF designed and performed brain tissue experiments and wrote the manuscript. CC performed all the cellular assays and participated in writing the manuscript. WW performed data extraction and bioinformatics analysis, and contributed to discussions and writing of the manuscript. BR ran the mass spectrometers and contributed to the progress in experimental setups. PT-O and CS-M helped with CD4^+^ T cell expansion. TZ performed immunohistochemistry and excision of brain tissue samples. MP analyzed MRI images and clinical data. IJ helped with analyzing the data and revised the manuscript. NS-W provided the characterized brain tissue samples. MS and RM were responsible for designing the questions of the study, acquisition of grants, and contributed to writing the manuscript.

### Conflict of Interest Statement

MP reports grants and personal fees from Novartis, Almirall, Biogen Idec, Sanofi Genzyme, and Teva outside the submitted work. He served as advisory board member of Novartis, Biogen, and Sanofi Genzyme. The remaining authors declare that the research was conducted in the absence of any commercial or financial relationships that could be construed as a potential conflict of interest.

## Supplementary Material

Figure S1 FDR (vertical axis) vs. score (horizontal axis). Green line FDR for deamidated and citrullinated peptides. Yellow line. FDR for all the other peptides.

Figure S2 Schematic picture of peptide fractionation and tissue sampling on HILIC.

Supplementary Table 1 Patient information: List of all patient tissues used in the study including tissue block numbers for proteomics studies.

Supplementary Table 2 List of peptides and quantification in control and MS brain tissues. The table includes the complete list of peptides used in the assays and the number of equivalent tryptic peptides found in the brain tissues of controls (C) and MS cases using LC-mass spectrometry (LC-MS) technique.

Supplementary Table 3 Extended clinical, including demographic and HLA-DRB1 haplotypes of MS patients. Gender, age, disease duration at LP, interval between last relapse and LP (Last relapse-LP delay), presence of ongoing clinical relapse (Clinically Active), the delay between MRI and LP (MRI-LP delay), presence of gadolinium-enhancing lesion at T1-MRI sequence (Radiologically active), IgG index, IgGOB Pattern, IgM Index, IgA index, and CSF cells count (/uL) and HLA-DRB1 typing are reported for each individual. CIS, clinical isolated syndrome; RRMS, relapsing remitting MS; OCB, oligoclonal bands.

Supplementary Data 1 All peptides identified with Mascot and Ursgal. PSM of each peptides with reference to search engines used and their tissue distribution.
