# Supplementary figures and images for: Brain Citrullination Patterns and T Cell Reactivity of Cerebrospinal Fluid-Derived CD4+ T Cells in Multiple Sclerosis
Source: Front Immunol. 2019 Apr 10;10:540. doi: 10.3389/fimmu.2019.00540 (PMC6467957; doi:10.3389/fimmu.2019.00540)

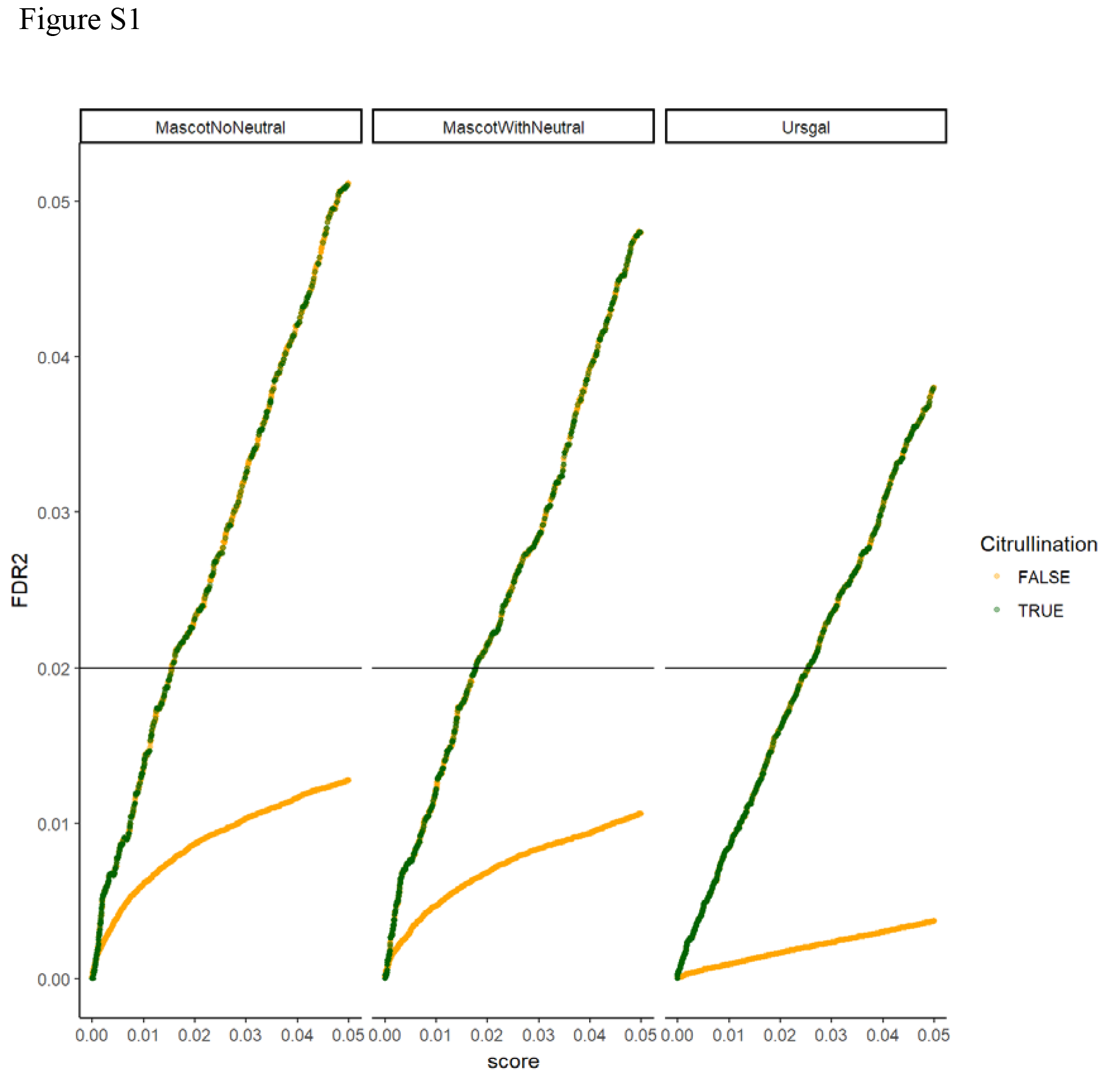

Supplement: Figure S1 — FDR (vertical axis) vs. score (horizontal axis). Green line FDR for deamidated and citrullinated peptides. Yellow line. FDR for all the other peptides. [file Image_1.tif]

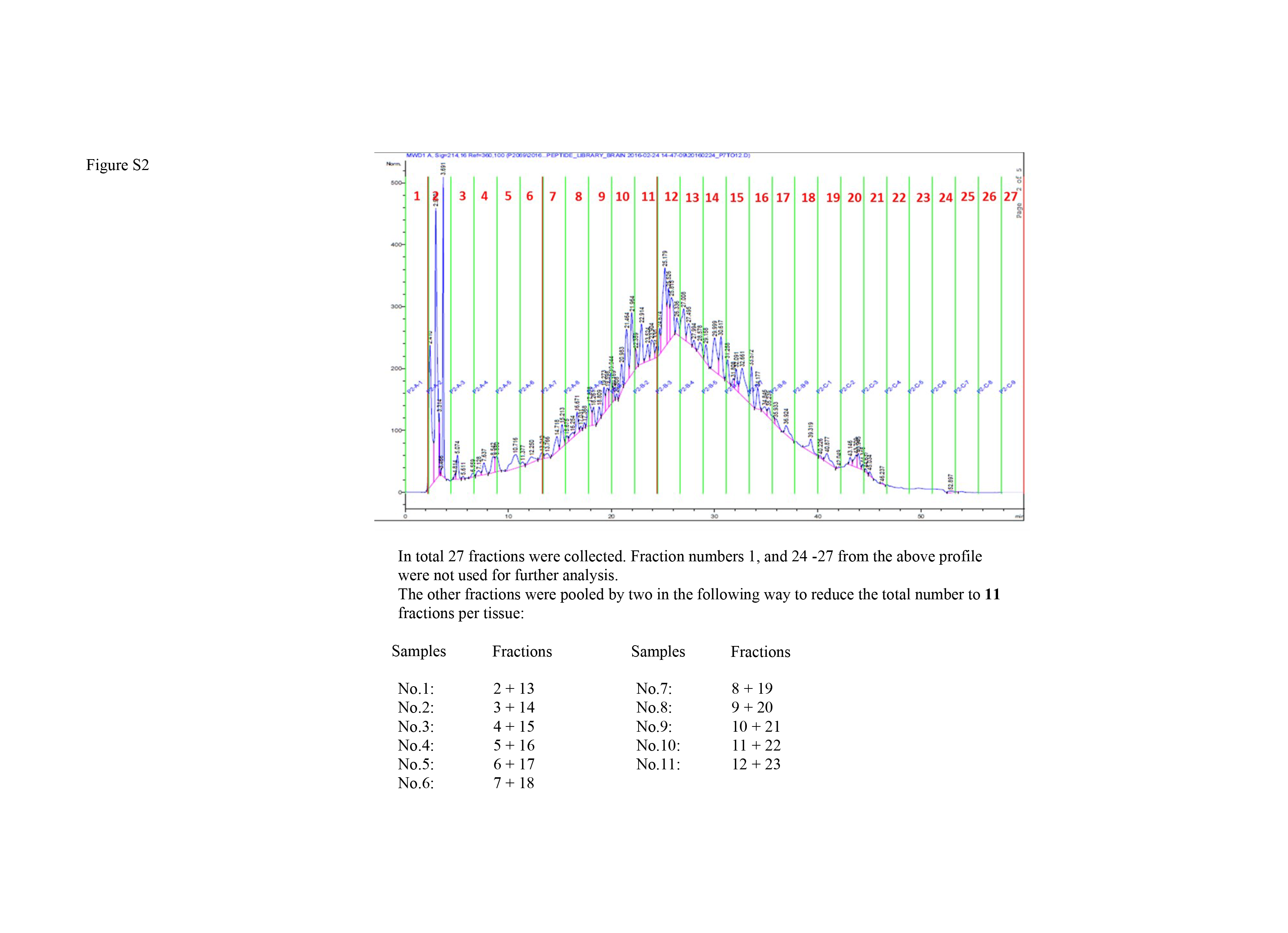

Supplement: Figure S2 — Schematic picture of peptide fractionation and tissue sampling on HILIC. [file Image_2.tif]
