# Supplementary material for: Brain Citrullination Patterns and T Cell Reactivity of Cerebrospinal Fluid-Derived CD4+ T Cells in Multiple Sclerosis
Source: Front Immunol. 2019 Apr 10;10:540. doi: 10.3389/fimmu.2019.00540 (PMC6467957; doi:10.3389/fimmu.2019.00540)
Supplement: Supplementary Table 1 — Patient information: List of all patient tissues used in the study including tissue block numbers for proteomics studies. [file Data_Sheet_1.PDF]

Supplementary table 1

Sample No.	Patient	Gender	Age	Cause of death	Post-mortem time (h)	Disease Duration (y)	MS type	Tissue
1	C18-3	M	35	Carcinoma of the tongue	22			GM
2	C28-2	F	60	heart failure, heart mestastasis	21			GM
3	C39-5	M	na.	Acute cardial death	10			GM
4	C21-1	M	75	Aspiration pneumonia	17			GM
5	C46-1	M	68	Metastatic colon cancer	10			GM
6	C30-1	M	69	pericardial tamponade	7			GM
7	M23-5	F	78	Metastatic carcinoma of bronchus	5	42	SPMS	GM
8	M32-3	F	39	Bronchopneumonia	18	21	PRMS	GM
9	M40-5	M	40	Respiratory failure, sepsis	10	9	SPMS	GM
10	M1-6	F	56	Breast carcinoma, Pneumothorax	8	31	SPMS	GM
11	M47-2	M	37	Intestinal obstruction	12	27	PPMS	GM
12	M14-2	F	78	Lung infection	9	47	PPMS	GM
13	C21-2	M	75	Aspiration pneumonia	17			WM
14	C15-5	M	64	Cardiac failure	18			WM
15	C28-2	F	60	Cardiac failure	21			WM
16	M51-2	F	49	Bronchopneumonia	12	27	PPMS	WM
17	M8-1	M	40	Dehydration / Multiple Sclerosis	18	23	SPMS	WM
18	M28-1	F	54	Bronchopneumonia	22	20	SPMS	WM
19	M6-7	F	58	Bronchopneumonia	6	21	PPMS	WM
20	M15-2	F	51	Multiple Sclerosis	15	21	SPMS	WM
21	M23-1	F	78	Metastatic carcinoma of bronchus	5	42	SPMS	WM
22	M2-9	F	58	Peritonitis, inflamed caecal diverticulum	16	22	PPMS	WM
23	M3-1	F	78	Myocardial infection, acute abdomen	18	33	SPMS	WM
24	M8-3	M	40	Dehydration / Multiple Sclerosis	18	23	SPMS	WM
