# Supplementary material for: Brain Citrullination Patterns and T Cell Reactivity of Cerebrospinal Fluid-Derived CD4+ T Cells in Multiple Sclerosis
Source: Front Immunol. 2019 Apr 10;10:540. doi: 10.3389/fimmu.2019.00540 (PMC6467957; doi:10.3389/fimmu.2019.00540)
Supplement: Supplementary Table 2 — List of peptides and quantification in control and MS brain tissues. The table includes the complete list of peptides used in the assays and the number of equivalent tryptic peptides found in the brain tissues of controls (C) and MS cases using LC-mass spectrometry (LC-MS) technique. [file Data_Sheet_2.PDF]

Supplementary table 2

Peptide name	**Peptide origin**	**Epitope desicion**	**Sequence**	**Citrullinated**	**# Tryptic Peptides**	
					**C**	**MS**
MBP-C(20-40)	Myelin Basic Protein	proteome study	T MDHA-**Cit-**HGFLP-**Cit-**H-**Cit-**DTGILDS	yes	184	206
MBP-C(38-56)	Myelin Basic Protein	proteome study	LDS IG-**Cit**-FFGGD-**Cit**-G APKRGS	yes	21	21
MBP-C(61-82)	Myelin Basic Protein	proteome study	HHPA-**Cit-**TAHYG SLPQKSHG-**Cit-**T QD	yes	84	84
MBP-C(83-106)	Myelin Basic Protein	proteome study	ENPVVHFF KNIVTP-**Cit-**TPP PSQGKG	yes	310	434
MBP-C(94-117)	Myelin Basic Protein	proteome study	VTP-**Cit-**TPP PSQGKGRGLS LS-**Cit-**FSWG	yes	310	434
MBP-C(108-126)	Myelin Basic Protein	proteome study	GLS LS-**Cit-**FSWGAEG Q-**Cit-**PGFG	yes	14	14
MBP-C(115-135)	Myelin Basic Protein	proteome study	SWGAEG Q-**Cit**-PGFGYGG-**Cit**-ASDYK	yes	35	35
MBP-C(146-170)	Myelin Basic Protein	proteome study	AQGTL SKIFKLGG-**Cit**-D S-**Cit**-SGSPMAR-**Cit**	yes	180	180
MBP-R(20-40)	Myelin Basic Protein	proteome study	T MDHA-**R**-HGFLP-**R**-H-**R**-DTGILDS	no	184	138
MBP-R(38-56)	Myelin Basic Protein	proteome study	LDS IG-**R**-FFGGD-**R**-G APKRGS	no	103	62
MBP-R(61-82)	Myelin Basic Protein	proteome study	HHPA-**R-**TAHYG SLPQKSHG-**R-**T QD	no	56	28
MBP-R(83-106)	Myelin Basic Protein	proteome study	ENPVVHFF KNIVTP-**R**-TPP PSQGKG	no	278	206
MBP-R(94-117)	Myelin Basic Protein	proteome study	VTP-**R**-TPP PSQGKGRGLS LS-**R-**FSWG	no	278	206
MBP-R(108-126)	Myelin Basic Protein	proteome study	GLS LS-**R-**FSWGAEG Q-**R-**PGFG	no	27	27
MBP-R(115-135)	Myelin Basic Protein	proteome study	SWGAEG Q-**R-**PGFGYGG-**R** ASDYK	no	175	140
MBP-R(146-170)	Myelin Basic Protein	proteome study	AQGTL SKIFKLGG-**R-**D S-**R-**SGSPMAR-**R**	no	135	90
MBP-C(13-32)	Myelin Basic Protein	immunodominance	KYLATASTMDHA-**Cit**-HGFLP-**Cit**-H	yes	0	22
MBP-C (83-99)	Myelin Basic Protein	immunodominance	ENPVVHFFKNIVTP-**Cit**-T	yes	310	434
MBP-C(111-129)	Myelin Basic Protein	immunodominance	LS-**Cit**-FSWGAEGQ-**Cit**-PGFGYGG	yes	105	129
MBP-C(146-170)	Myelin Basic Protein	immunodominance	AQGTLSKIFKLGGRDS-**Cit**-SGSPMAR-**Cit**	yes	324	333
MBP-R(13-32)	Myelin Basic Protein	immunodominance	KYLATASTMDHA-**R**-HGFLP-**R**-H	no	184	138
MBP-R(83-99)	Myelin Basic Protein	immunodominance	ENPVVHFFKNIVTP-**R**-T	no	4	4
MBP-R(111-129)	Myelin Basic Protein	immunodominance	LS-**R**-FSWGAEGQ-**R**-PGFGYGG	no	202	167
MBP-R(146-170)	Myelin Basic Protein	immunodominance	AQGTLSKIFKLGGRDS-**R**-SGSPMAR-**R**	no	135	90
PLP-(139- 154)	Proteolipid Protein	immunodominance	HCLGKWLGHPDKFVGI	no	3	0
MOG-(1-20)	Myelin Oligodendrocyte Glycoprotein	immunodominance	GQF-**R**-VIGP-**R**-HPI-**R**-ALVGDEV	no	3	0
MOG-(35-55)	Myelin Oligodendrocyte Glycoprotein	immunodominance	MEVGWY-**R**-PPFS-**R**-VVHLY-**R**-NGK	no	91	79
