# Supplementary material for: Brain Citrullination Patterns and T Cell Reactivity of Cerebrospinal Fluid-Derived CD4+ T Cells in Multiple Sclerosis
Source: Front Immunol. 2019 Apr 10;10:540. doi: 10.3389/fimmu.2019.00540 (PMC6467957; doi:10.3389/fimmu.2019.00540)
Supplement: Supplementary Table 3 — Extended clinical, including demographic and HLA-DRB1 haplotypes of MS patients. Gender, age, disease duration at LP, interval between last relapse and LP (Last relapse-LP delay), presence of ongoing clinical relapse (Clinically Active), the delay between MRI and LP (MRI-LP delay), presence of gadolinium-enhancing lesion at T1-MRI sequence (Radiologically active), IgG index, IgGOB Pattern, IgM Index, IgA index, and CSF cells count (/uL) and HLA-DRB1 typing are reported for each individual. CIS, clinical isolated syndrome; RRMS, relapsing remitting MS; OCB, oligoclonal bands. [file Data_Sheet_3.PDF]

Supplementary table 3

PATIENT	DISEASE	GENDER	Age	Disease Duration at LP (months)	Last relapse-LP delay (months)	Clinically Active	MRI-LP delay (months)	Radiologically active	IgG index	IgGOCB	IgM Index	IgA index	Cells/uL	DRB1 Allele1	DRB1 Allele 2
**1444ME**	RRMS	F	30	0	0	yes	0	no	1.08	CSF-restricted	0.05	0.27	7	07:01	13:02
**1440AM**	RRMS	M	24	6	6	no	0	no	0.67	CSF-restricted	0.83	0.33	2	08:01	13:02
**1460 ML**	RRMS	F	36	0	0	yes	0	no	0.64	CSF-restricted	0.07	0.23	2	03:01	15:01
**1673UR**	CIS	M	47	2	2	no	0	no	0.48	Mirror Pattern	0.04	0.38	2	04:01	11:04
**1188ZA**	RRMS	F	43	0	0	yes	3	no	0.74	CSF-restricted	0.06	0.28	6	01:01	11:04
**1283RO**	RRMS	M	17	32	0	no	0	yes	0.56	CSF-restricted	0.19	0.31	-	10:01	13:03
**1346JU**	RRMS	F	21	1	1	yes	0	yes	1.08	CSF-restricted	0.08	0.32	12	07:01	13:01
**1517PH**	RRMS	M	40	0	0	yes	0	yes	0.74	CSF-restricted	0.06	0.30	4	15:01	15:01
**1479CR**	RRMS	F	28	2	2	no	0	yes	1.61	CSF-restricted	0.38	0.36	6	13:01	13:02
**697NU**	RRMS	F	19	0	0	no	-2	no	0.89	CSF-restricted	0.23	0.29	9	12:01	13:03
**1560RO**	PPMS	M	56	65	n.a.	no	-1	no	0.75	CSF-restricted	0.07	0.24	1	07:01	15:01
**1664SI**	RRMS	M	31	14	2	no	0	yes	0.55	CSF-restricted	0.48	0.26	7	04:04	15:01
**996NM**	RRMS	F	31	0	0	yes	0	yes	1.14	CSF-restricted	0.08	0.30	5	07:01	15:01
**1467CL**	RRMS	F	33	0	0	yes	0	yes	2.08	CSF-restricted	0.59	0.35	8	03:01	15:01
**1458 MI**	RRMS	F	55	241	0	yes	0	no	0.52	CSF-restricted	0.05	0.36	10	12:01	15:02
**474MS**	RRMS	F	27	30	1	yes	0	yes	0.55	CSF-restricted	0.25	0.33	7	11:04	07:01
**1453AN**	RRMS	M	49	2	2	no	-1	no	0.83	CSF-restricted	0.27	0.46	10	04:02	11:01
**1586NI**	RRMS	F	43	1	1	yes	0	yes	0.94	CSF-restricted	0.28	0.33	10	03:01	15:01
**1493 MO**	PPMS	F	45	13	n.a.	no	-1	yes	1.13	CSF-restricted	0.03	0.23	4	03:01	07:01
**1489 HE**	RRMS	F	58	8	8	no	0	no	0.78	CSF-restricted	0.07	0.29	3	10:01	12:01
**1371MS**	RRMS	F	38	0	0	yes	0	yes	2.51	CSF-restricted	0.19	0.24	38	01:02	13:03
**1515 MA**	RRMS	F	50	1	1	no	-1	no	0.79	CSF-restricted	0.03	0.23	4	13:01	15:01
